# Long-read, high-coverage reference genome of the nymphalid butterfly *Catonephele acontius* (Nymphalidae: Biblidinae)

**DOI:** 10.1093/g3journal/jkag174

**Published:** 2026-07-02

**Authors:** Marcus Hicks, Tan Nhat Pham, Océane Seudre, Zunilda Escalante-Arteaga, Lucy S. Knowles, Geoffrey Gallice, Vicencio Oostra

**Affiliations:** School of Biological and Behavioural Sciences, Queen Mary University of London, London, Greater London E1 4NS, United Kingdom; NERC Environmental Omics Facility, NEOF Visitor Facility, School of Biosciences, University of Sheffield, Alfred Denny Building, Western Bank, Sheffield S10 2TN, United Kingdom; Department of Systematic Zoology, Faculty of Biology, Institute of Environmental Biology, Adam Mickiewicz University, Poznan, Greater Poland 61-712, Poland; Vietnam Forest Museum, Forest Inventory and Planning Institute, Hanoi 11707, Vietnam; School of Biological and Behavioural Sciences, Queen Mary University of London, London, Greater London E1 4NS, United Kingdom; Alianza Para una Amazonía Sostenible Perú, Las Piedras, Madre de Dios 17100, Perú; NERC Environmental Omics Facility, NEOF Visitor Facility, School of Biosciences, University of Sheffield, Alfred Denny Building, Western Bank, Sheffield S10 2TN, United Kingdom; Departamento de Ingeniería, Pontificia Universidad Católica del Perú, Lima 15088, Perú; Alliance for a Sustainable Amazon, Potomac, MD 20854, United States; Department of Natural History, Florida Museum of Natural History, University of Florida, Gainesville, FL 32611, United States; School of Biological and Behavioural Sciences, Queen Mary University of London, London, Greater London E1 4NS, United Kingdom

**Keywords:** *de novo* genome, neotropics, Lepidoptera, PacBio, Amazonía, comparative genomics, climate, diapause, genome assembly

## Abstract

*Catonephele acontius* (Nymphalidae:Biblidinae:Epicalinii) is a butterfly species with a wide distribution across the Neotropics including the Amazon. Here, we present a long-read high-coverage reference genome for this species to serve as a genomic resource for future studies on Biblidinae butterflies, a group that is the subject of ongoing studies of seasonal adaptation under climate change. We used PacBio HiFi and IsoSeq reads to generate a highly contiguous and well-annotated reference genome. Five libraries were constructed, 4 using RNA from different tissues and 1 using high molecular weight (HMW) DNA from a wild-caught female. The DNA was sequenced using PacBio HiFi technology, and the RNA was sequenced using long read PacBio IsoSeq technology. About 20 Gb of raw HiFi data were generated and assembled to an initial size of 520.7 Mb (39 × homozygous coverage) in 90 contigs. The assembly was then polished and decontaminated into 40 contigs with an N50 of 19.927 Mb (BUSCO completeness: 99.0%; duplication: 0.5%; fragmentation: 0.7%; and missing: 0.3%). Final assembly size was 519.2 Mb. Repeats were annotated, showing that the genome consisted of 40.4% transposable elements. IsoSeq transcriptome data from antennae, leg, ovary, and digestive tissue was then used to structurally and functionally annotate gene models for the softmasked genome, uncovering ∼18,500 genes, with 70% of them given functional annotation. This reference assembly joins many published genomes in the Nymphalidae family but represents one of the first high-quality genomes from the Biblidinae subfamily. It provides a valuable resource to study the evolution of plastic and seasonal traits and will help investigate the genetic processes that may influence these species' responses to rapid climate change.

## Introduction

Lepidoptera are important models in evolutionary and ecological studies, and key biodiversity indicators. Recently, there has been a rapid and impressive increase in the number of Lepidoptera reference genomes (e.g. Wright et al. 2025). Nevertheless, species from the tropics remain severely underrepresented in genomic databases, including in the Neotropics (Shirey et al. 2022), and important phylogenetic gaps remain. The subfamily Biblidinae is a diverse group of butterflies comprising more than 300 species distributed across Central and South America. As part of a wider effort to document their ecology, evolutionary history, and life history strategies, the first high-quality *de novo* genome for this subfamily was recently sequenced (Pham et al. 2025).

Well-resolved *de novo* genome assemblies have become increasingly accessible with the availability of long-read sequencing technologies and support a wide variety of genomic analyses, especially when complemented with RNA-seq data from diverse tissues yielding well-supported gene models. Such highly contiguous genomes enable, for example, coalescent-based demographic analyses (Rosenberg and Nordborg 2002, Cousins et al. 2025), the study of long transposable elements (Makałowski et al. 2019), and large-scale comparative genomics analyses (e.g. Christmas et al. 2023). Well-annotated genomes are critical for gene expression studies (e.g. Bloch et al. 2021) when focusing on alternative splicing (e.g. Steward et al. 2022) and epigenetic analyses especially when used alongside ATAC-seq data (Ruggieri et al. 2022). In tandem with Hi-C data, chromosome-level assemblies further allow for comparative genomics analyses focusing on the evolution of chromosome number across a phylogeny (Wright et al. 2024).

**Fig. 1.**
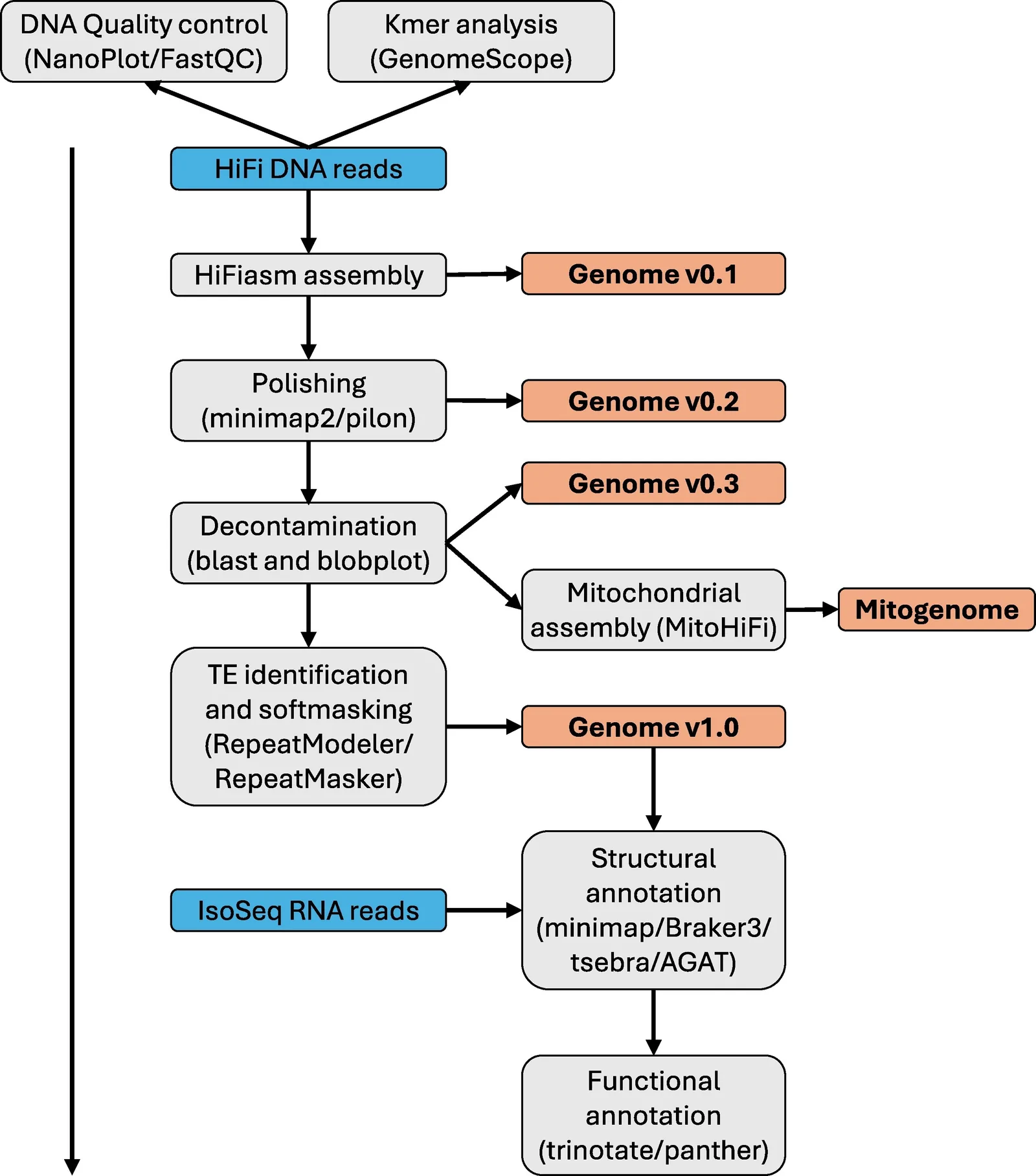
Overview of the Genome assembly and annotation stages, highlighting the 3 versions created, from v0.1 (primary assembly), v0.2 (polished assembly) and v0.3 (decontaminated assembly). v1.0 refers to the final, softmasked genome.

Here, we provide a high-quality draft nuclear and mitochondrial genome assembly and annotation for *Catonephele acontius* (ilCatAcon1) (Linnaeus 1771) (Lepidoptera: Nymphalidae: Biblidinae) (Fig. 1). This species has a wide distribution in Central and South America. Previous studies on its life history documented an approximate 2-month lifespan in butterfly houses (Gonzalez 2024), and its likely host plant is *Alchornea latifolia* (Euphorbiaceae), (Alberto Muyshondt 1975; Beccaloni et al. 2008). More recently, we have developed this species as a model to study adult reproductive plasticity in seasonal environments, where egg production differs between the wet and dry season (Hicks et al. 2026). Here, we generate a *de novo* reference genome for this species. We combine high coverage PacBio Hifi DNA data with PacBio IsoSeq RNA data for 4 different adult tissues, enabling comprehensive gene and repeat annotation. We also compare the assembly to that of *Melitaea cinxia*, a nymphalid butterfly with a high-quality reference genome.

This new reference genome will serve as a valuable resource in the study of Biblidinae and Lepidoptera in general, particularly for emerging research on reproductive strategies, migration, and seasonal plasticity in this tribe (Hicks et al. 2026).

## Materials and methods

### Sampling strategy

One *C. acontius* female was caught using rotten banana-baited traps in terra firme lowland Amazon rainforest (Finca Las Piedras field station in Madre de Dios, SW Peru lat. −12.226348°, lon. −69.112599, elevation 270 m) in September 2023 (during the dry season) and was chosen at random from a long-term collection effort at the site. The individual butterfly was identified based on well-defined interspecific differences in wing patterns following a field guide (Warren et al. 2012), which includes reliable characters for species identification in the genus *Catonephele* (Supplementary Fig. 1). The specimen was stored in RNAlater (Invitrogen) at room temperature, transferred to −10 °C the same day for 30 d, transported at ambient temperature to the UK before being stored long term at −80 °C (Supplementary Table 1).

### Sample preparation and sequencing methods

Genomic DNA was extracted from the thorax of the *C. acontius* female at the NERC Environmental Omics Facility (NEOF) using the Macherey-Nagel Nucleobond HMW DNA Kit. Lysis buffer, proteinase K volume, and binding buffer were doubled, compared to the kit's protocol, and all remaining steps were carried out according to the manufacturer's instructions. RNA was extracted from 5 tissues (legs, antennae, reproductive tissue, digestive tract, and head) from the same individual as the genomic DNA using a QIAGEN RNeasy Mini Kit according to the manufacturer's instructions, with an extra wash of the RNeasy spin column with buffers RPE and RW1. The quantity and purity of the extracted DNA and RNA were measured using a Qubit fluorometer and NanoDrop (both Thermo Fisher). Integrity of the DNA was assessed using a Femto Pulse and that of the RNA using a TapeStation (both Agilent). The RNA from the head tissues had poor integrity and was likely contaminated (NanoDrop 260:230 < 1; heavily colored) so was discarded. DNA and RNA samples were sent to the Center for Genomic Research at the University of Liverpool (CGR) where 5 libraries (1 DNA and 4 RNA) were constructed. cDNA synthesis was performed on the RNA samples using the PacBio Iso-Seq Express 2.0 Kit and libraries were constructed for both cDNA and DNA samples using the PacBio SMRTbell prep kit 3.0. The PacBio HiFi DNA library was sequenced at CGR on a SMRT PacBio cell, generating long HiFi reads with a total output of 19.9Gbp (Wenger et al. 2019). PacBio IsoSeq RNA libraries were sequenced at CGR on 1 SMRT IsoSeq PacBio cell with an output range of 46.8 to 67.6 Mbp. NanoPlot (De Coster and Rademakers 2023) was used to produce the sequencing output for all libraries shown in Table 1. We calculated mean read lengths for RNA libraries using samtools v1.10 (Li et al. 2009).

**Table 1.** Sequencing output of PacBio HiFi (DNA) and IsoSeq (RNA) libraries.

Library type	Mean read length (kb)	Number of raw reads	Number of bases
Thorax DNA (HiFi)	17.27	1,152,949	19,912,358,802
Ovary RNA (IsoSeq)	2.12	392,033	46,807,863
Legs RNA (IsoSeq)	2.07	540,102	65,825,088
Antennae RNA (IsoSeq)	2.04	567,721	67,596,791
Digestive tissue RNA (IsoSeq)	2.15	408,458	50,227,001

For the DNA library, kmers (k = 31) were counted using FastK (Myers Eugene 2021). We assessed frequency distributions and genome characteristics using GenomeScope v2.0.1 (Vurture et al. 2017, Ranallo-Benavidez et al. 2020) (Fig. 3). We used a range of k-values (21, 31, and 41) and another program Jellyfish v2.3.1 (Marçais & Kingsford, 2011), which resulted in similar genome size estimates (Supplementary Table 8).

### Nuclear genome assembly

Initial genome assembly was performed using Hifiasm v0.19.9 (Cheng et al. 2021) using default parameters, including purging duplicates (parameter -l 3: to purge all types of haplotigs in the most aggressive way; parameter -s 0.55: a similarity threshold for duplicate haplotigs that should be purged). Genome statistics were computed using the stats package of bbmap v39.34 (Supplementary Tables 2 and 3) (Bushnell 2014). To polish the primary assembly (v0.1), we first aligned the raw HiFi reads back to genome v0.1 using Minimap2 v2.17 (Li, 2018) and converted the results to a BAM file using samtools v1.10 (Li et al. 2009). We then polished the primary contigs with the raw HiFi reads using Pilon to create v0.2 (Walker et al. 2014).

For decontamination resulting in genome v0.3, we used BlobToolKit v1.1.1 (Challis et al. 2020), which assesses GC content and coverage statistics for each primary contig and infers taxonomic assignments (Fig. 4).

To determine chromosomal-level completeness, we use the TeloExplorer module implemented in quarTeT 1.2.5 (Lin et al. 2023) to find putative telomeric repeats, with a minimum cutoff of 10 repeats.

### Mitochondrial genome assembly

**Fig. 2.**
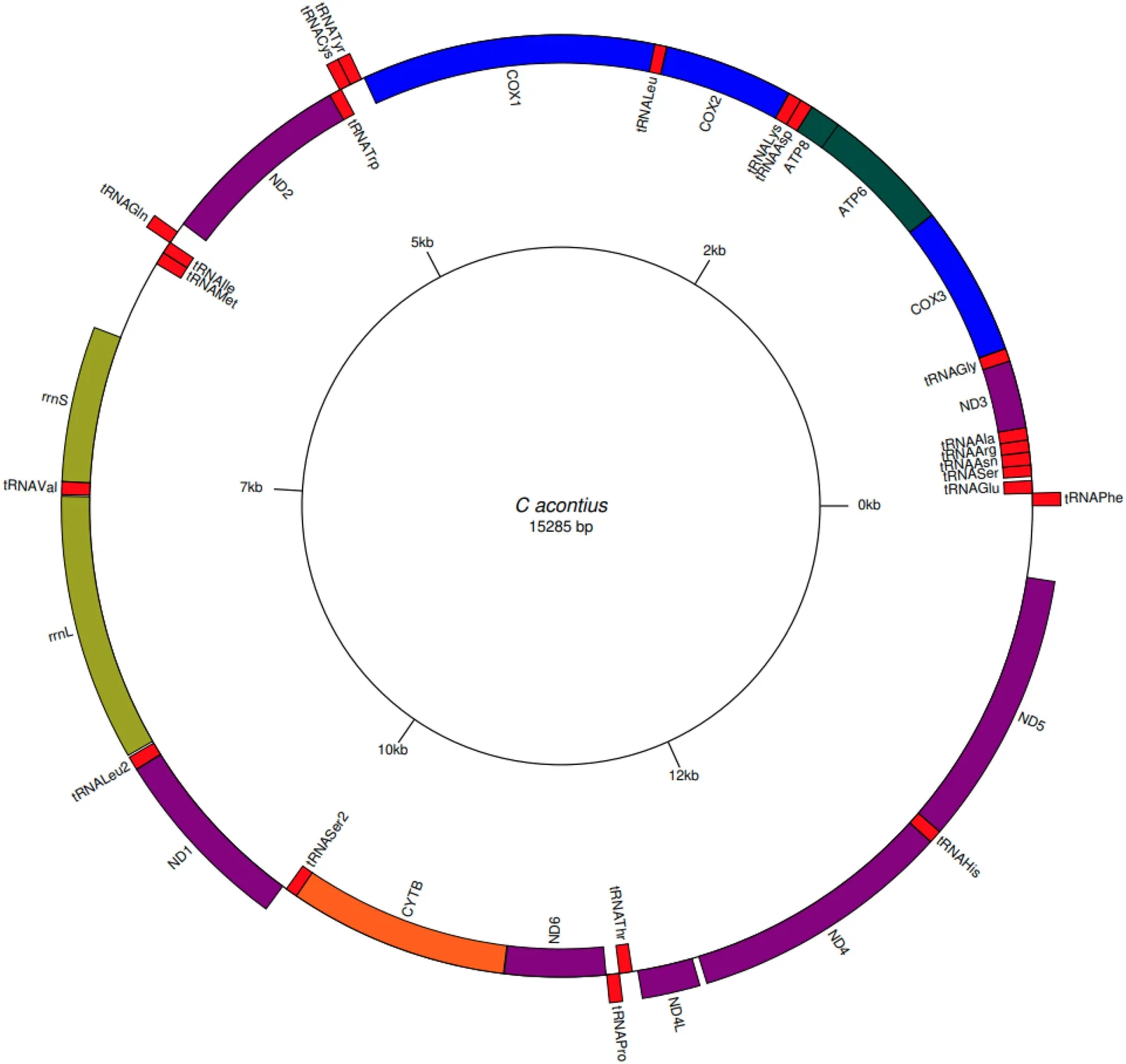
Annotated circular mitogenome for *Catonephele acontius*. It shows the arrangement of labeled protein-coded genes, ribosomal RNAs and transfer RNAs. Annotation on the inner/outer circles indicate orientation (−/+). To aid visualization, the gene order (anti-clockwise from 0 kb) is as follows: (1) tRNA-Phe, (2) tRNA-Glu, (3) tRNA-Ser, (4) tRNA-Asn, (5) tRNA-Arg, (6) tRNA-Ala, (7) ND3, (8) tRNA-Gly, (9) COX3, (10) ATP6, (11) ATP8, (12) tRNA-Asp, (13) tRNA-Lys, (14) COX2, (15) tRNA-Leu, (16) COX1, (17) tRNA-Tyr, (18) tRNA-Cys, (19) tRNA-Trp, (20) ND2, (21) tRNA-Gln, (22) tRNA-Ile, (23) tRNA-Met, (24) rrnS, (25) tRNA-Val, (26) rrnL, (27) tRNA-Leu2, (28) ND1, (29) tRNA-Ser2, (30) CYTB, (31) ND6, (32) tRNA-Pro, (33) tRNA-Thr, (34) ND4L, (35) ND4, (36) tRNA-His, (37) ND5.

The mitochondrial genome was assembled using MitoHiFi v3.2.2 and annotated using MitoFinder v1.4.2 (Figure 2) (Allio et al. 2020; Uliano-Silva et al. 2023). We subsequently re-annotated the mitogenome *de novo* using MITOS2 (using invertebrate mitochondrial code (5) with RefSeq63 Metazoa reference data and default parameters) (Al Arab et al. 2017; Donath et al. 2019) after noticing a missing gene annotation (ATP8) from the initial MitoHiFi annotation. We created the circular mitogenome visualizations (Fig. 2) using GenomeVx (Conant and Wolfe 2008). MitoFinder also identified contigs that were potentially mitochondrial in origin. To test whether these were indeed mitochondrial contaminants of the nuclear genome assembly, we blasted the mitochondrial genome against all other contigs in the nuclear genome assembly using Blast + v2.17.0 (Zhang et al. 2000; Camacho et al. 2009). Considering the bit score, % identity and coverage of this blast across the subject contig, as well as the corresponding size (<35 kbp) and GC content (<0.25) of all the contigs (Supplementary Table 9), we removed 50 small contigs from the nuclear genome assembly that all appeared to be mitochondrial, creating genome v0.3 with 40 contigs whilst maintaining the same, high BUSCO completeness score of 99.0, respectively as well as low duplication (0.5%), fragmentation (0.7%) and missing (0.3%) scores (Table 2).

**Table 2.** Genome completeness (BUSCO) and contiguity statistics for subsequent genome versions.

Genome	Genome size (Mb)	Number of contigs	GC%	Complete BUSCOs *N* (%)	Complete and single-copy *N* (%)	Complete and duplicated *N* (%)	Fragmented *N* (%)	Missing *N* (%)	N50 (Mbp)	L50 (contigs)	L90 (contigs)	Percentage of Ns
*C. acontius* v0.1	520.701	90	33.32	5230 (99.0)	5205 (98.5)	25 (0.5)	37 (0.7)	19 (0.3)	19.928	11	23	0
*C. acontius* v0.2	520.207	90	33.31	5231 (99.0)	5207 (98.5)	24 (0.5)	36 (0.7)	19 (0.3)	19.927	11	23	0
*C. acontius* v0.3	519.245	40	33.34	5231 (99.0)	5207 (98.5)	24 (0.5)	36 (0.7)	19 (0.3)	19.927	11	23	0
*C. acontius* v1	519.245	40	33.34	5231 (99.0)	5207 (98.5)	24 (0.5)	36 (0.7)	19 (0.3)	19.927	11	23	0

### Repeat assembly

RepeatModeler v2.0.6 (Flynn et al. 2020) and Repbase (Bao et al. 2015) were used to build a de novo library of repeats for the genome. To avoid misclassifying expanded gene families as transposable elements (TEs), the RepeatModeler consensus library was screened against the predicted protein set of *Melitaea cinxia* (Smolander et al. 2022) using DIAMOND BLASTP v2.1.11 (Buchfink et al. 2015) with an e-value cutoff of 1×10^−10^. Consensus sequences showing significant similarity to *bona fide* proteins were removed prior to downstream TE annotation.

Repeat consensus sequences were classified using TEclass (Abrusán et al. 2009). We next used RepeatMasker v4.1.7 (Smit et al. 2015) with the curated library and TEclass labels to annotate repeats and LTR-finder v1.07 (Xu and Wang 2007) to identify long terminal repeat retrotransposons. These results were integrated using RepeatCraft (Wong and Simakov 2019) to produce a consensus. Bedtools v2.31.1 (Quinlan 2014) was then used to soft mask the genome assembly.

Divergence landscapes were generated by estimating Kimura substitution levels with calcDivergenceFromAlign.pl and a custom-modified createRepeatLandscape.pl (published in (Moggioli et al. 2023)), both from RepeatMasker (Smit et al. 2015). Repeat landscapes and TE class composition were visualized in R (R Core Team 2021) using *ggplot2 v3.5.1* (Wickham, 2016) (Fig. 5).

### Gene prediction

PacBio IsoSeq transcript data were aligned to the softmasked genome using minimap2 v2.28 (Li, 2018) with splice-aware settings optimized for high-quality IsoSeq reads. Resulting alignments were sorted and indexed with SAMtools 1.19.2 (Danecek et al. 2021).

Gene prediction was performed with BRAKER3 v3.0.8 (Gabriel et al. 2024), in 2 complementary modes. First, IsoSeq alignments were used as extrinsic evidence, enabling alternative transcript prediction. Second, protein homology evidence was provided using the Arthropoda odb10 protein data set from OrthoDB (Kriventseva et al. 2019) supplemented with *Melitaea cinxia* proteins (Smolander et al. 2022). In both runs, BRAKER3 was executed with species-specific parameter settings in its default iterative training mode, whereby GeneMark-EP + v4.72 (Brůna et al. 2020) first generated initial gene models that were then used to retrain AUGUSTUS v3.5.0 (Stanke et al. 2006) prior to the final gene prediction. The 2 BRAKER3 runs were reconciled with TSEBRA v1.1.2.5 (Gabriel et al. 2021) using the standard BRAKER3 configuration, giving priority to IsoSeq-supported models for tie-breaking.

Finally, the gene set was filtered for longest isoforms and standardized with AGAT v.1.4.3 (Dainat 2024). In-frame stop codons were removed with gffreads v0.12.7 (Pertea and Pertea 2020) in “-V -H” mode. Genes overlapping annotated transposable elements were excluded using custom scripts published in study by Martín-Zamora et al. (2023). Additional spurious models showing high similarity to transposable element proteins were removed with DIAMOND BLASTP v2.1.11 (Buchfink et al. 2015) against the RepeatPeps.lib library, using an e-value cutoff of 1e-30. This threshold was selected after testing alternative cutoffs and evaluating their effects on BUSCO scores and gene counts (Supplementary Table 6).

Gene completeness and annotation quality were assessed at each step with BUSCO v5.8.2 (Manni et al. 2021) in protein mode against the “lepidoptera_odb10” database (*n* = 5193 BUSCOs) (Supplementary Table 6).

### Functional annotation

Functional evidence was assigned with Trinotate v4.0.2 (Bryant et al. 2017), which queried the final protein set against multiple sources: BLASTP and BLASTX v2.16.0 searches against the UniProtKB/SwissProt database (Boutet et al. 2007), Pfam domain detection with HMMER v3.3 (Potter et al. 2018), and signal peptide prediction with SignalP v5.0 (Teufel et al. 2022). In parallel, proteins were classified into curated families and subfamilies with PANTHER v19.0 (Thomas et al. 2003). Results from Trinotate and PANTHER were merged in R (R Core Team 2021) to produce a unified annotation table that served as the final functional dataset for downstream analyses.

A comparative synteny analysis was then carried out using dotplots comparing *Catonephele acontius* against the complete genome of *Melitaea cinxia*. We chose this species because it is a Nymphalid with a high-quality assembly and annotation. Dotplots were created in R using the following packages: *dplyr v1.1.4* (Wickham et al. 2014)*, ggplot2 v3.5.1* (Wickham, 2016)*, knitr v1.49* (Xie 2014)*, magrittr v2.0.3* (Bache and Wickham 2022)*, tidyr v1.3.1* (Wickham et al. 2024), and *stringr v1.6.0* (Wickham 2025).

## Results and discussion

### Genome details

The initial genome size (v0.1) was 520.7 Mb. It was comprised of 90 contigs of which the largest 23 contained 90% of the genome with an N50 value of 19.928 Mb. Gene content was highly complete for the primary assembly, v0.1, with a BUSCO completeness score of 99%, and duplication (0.5%), fragmentation (0.7%) and missing (0.3%) scores all low (Table 2) (Seppey et al. 2019). These low duplication rates are expected given aggressive purging of duplicates as part of Hifiasm. After polishing and decontamination, the final genome (v1.0) was 519.2 Mb while BUSCO completeness scores remained high (99.0%), and duplication (0.5%), fragmentation (0.7%) and missing (0.3%) scores remained low. We further assessed genomic content using the mapping rate of the IsoSeq data against the assembly and found very high mapping rates across all tissues (>99.8%) (Supplementary Table 4). The assembled mitogenome measured 15,285 bp and contained 37 genes (Fig. 2).

### Coverage and quality analysis

**Fig. 3.**
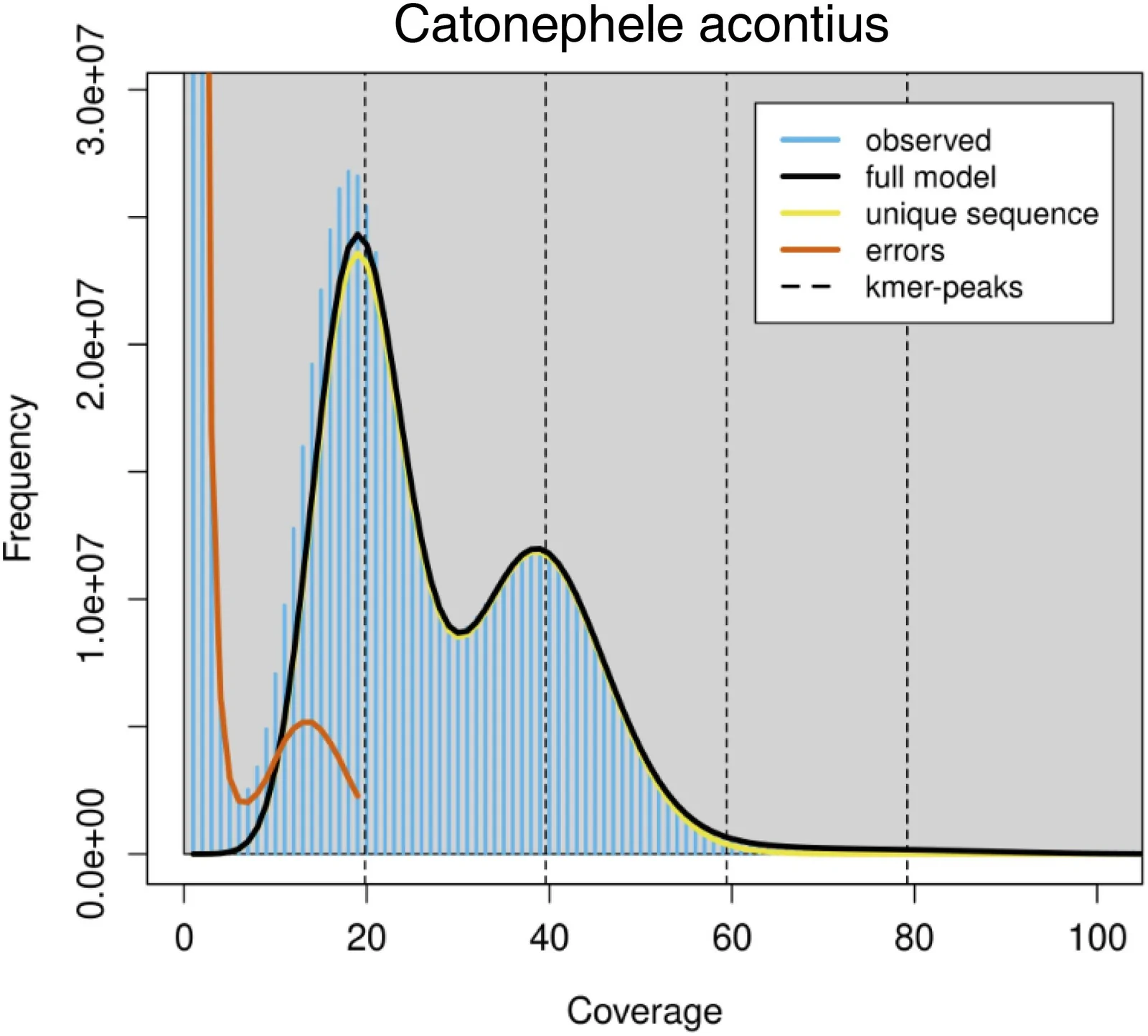
Kmer distribution analysis kmer frequency distribution (k = 31, ploidy = 2, coverage peaks at 20 × and 40 × – vertical dashed lines) and an estimated genome size of 416 Mbp (402–440 Mbp, Supplementary table 8) with heterozygosity of 1.7%. The blue bars are the observed distribution of Kmer coverage, whilst the black lines represent the full Kmer distribution model, the yellow line models only unique Kmers, and the red models error Kmers.

The heterozygosity estimate was 1.7% (Fig. 3), which sits within the expected range for non-migratory Lepidoptera (0.21%–3.62%) (García-Berro et al. 2023). There is a discrepancy between the estimated genome size from the kmer analyses (k = 31: ∼425 Mbp) and the final assembly size (∼520 Mbp). This finding was independent of kmer size (k = 21: 402 Mbp, k = 41: 418 Mbp using FastK, k = 31: 440 Mbp using Jellyfish, Supplementary Table 8) and of maximum kmer frequency filtering (<10,000,000). This may be because this genome is highly repetitive (∼45%) and has relatively high heterozygosity (1.7%), both of which are factors that can lead to underestimation of genome size by GenomeScope (Vurture et al. 2017). This was also observed in *Tirumala septentrionis*, a Danaid butterfly species with comparably high heterozygosity (1.74%) and repeats (32.6%), which also had its genome size underestimated (338 Mbp to 381.4 Mbp) compared with other similar genome assemblies with lower heterozygosity and repeats (Mora et al. 2024).

**Fig. 4.**
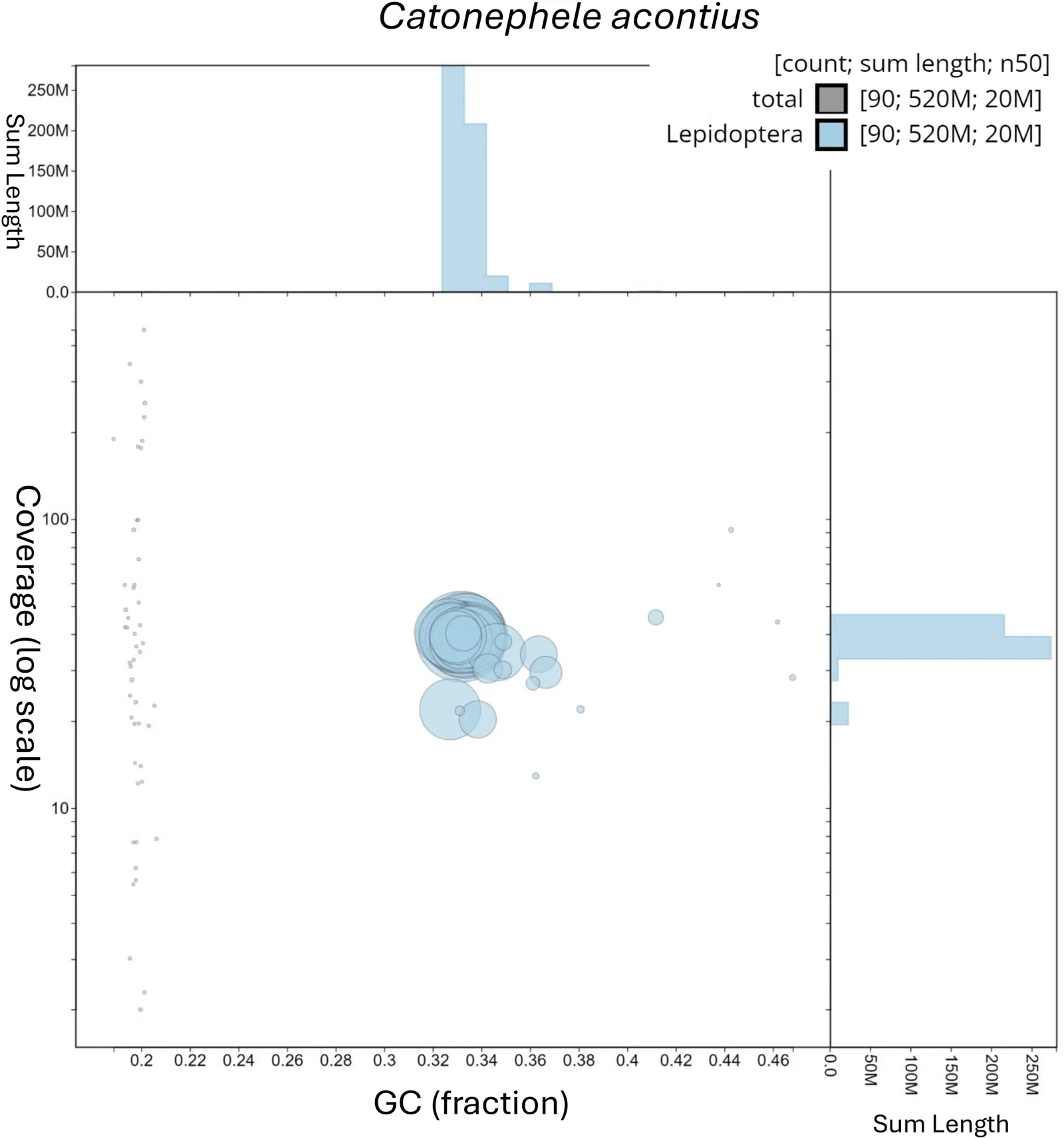
No non-lepidopteran contamination in genome v0.2. Blobplot showing the GC% and coverage for each contig (on a log scale), with the size of bubble representing the size of the contig. The small bubbles with low GC content are mitochondrial in origin, whilst the others are larger nuclear contigs. Full contig-level output can be found in Supplementary Table 11. We did not identify any putative *Wolbachia* sequence.

Our blast analysis within BlobToolKit (Challis et al 2020) showed that there was no non-lepidopteran contamination (Fig. 4) although some of the smaller contigs were mitochondrial in origin (Supplementary Table 9). We did not identify any putative *Wolbachia* sequence, in contrast to identification of *Wolbachia* in the *Batesia hypochlora* reference genome from the same Biblidinae subfamily (Pham et al. 2025).

### Analysis of repetitive DNA

Repetitive elements accounted for 44.40% of the genome. TEs accounted for 40.09% of the nuclear genome content (Supplementary Table 5). Such proportions are comparable to *B. hypochlora* in which 34% of the nuclear assembly is TEs (Pham et al. 2025). We found LINEs as the most abundant class, closely followed by DNA transposons, consistent with the TE composition reported for *B. hypochlora*, where LINEs predominate (Pham et al. 2025).

**Fig. 5.**
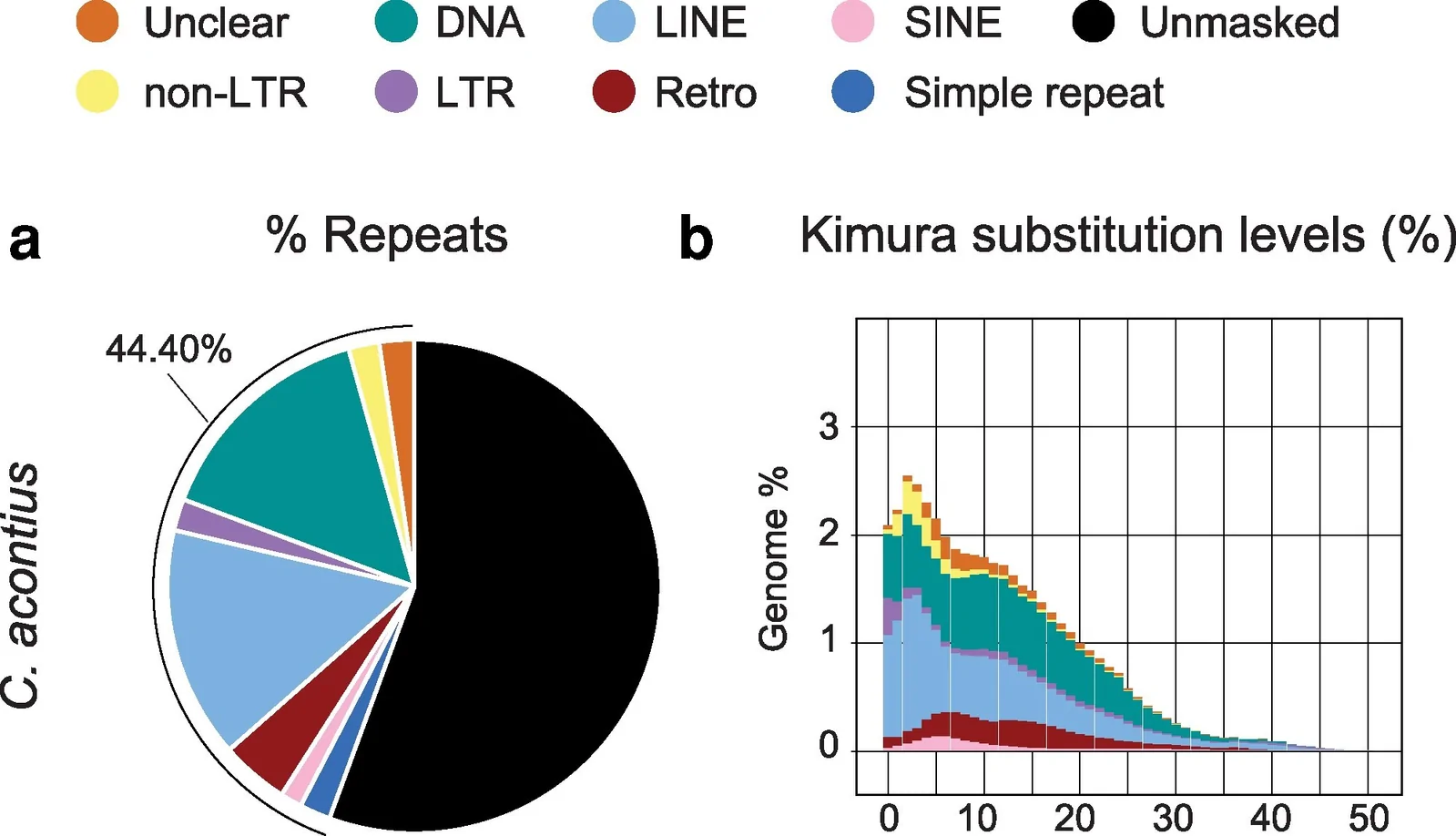
Repetitive landscape: a) pie charts showing proportions of repetitive elements. b) The distribution plot on the figure's right shows the Kimura substitution levels of repeat elements and their proportion in the genome. Supporting data can be found in Supplementary Table 5. LTR, long terminal repeats; LINE, long interspersed nuclear element; SINE, short interspersed nuclear element.

Most repeats showed low Kimura substitution levels (<25%), indicating a predominance of evolutionarily young elements while older repeats appear to be largely purged (Fig. 5). Kimura substitution levels measure the fraction of nucleotides in the repeat that have changed compared with the original consensus repeat. Low levels of substitution indicate a young repeat—high levels suggest an old repeat. High levels of recent DNA transposon activity have been seen in other Neotropical butterflies such as *Heliconius melpomene* (Lavoie et al. 2013) and may play a large role in their evolution and divergence (Ray et al. 2019).

### Genome annotation

Using our IsoSeq RNA data, we identified 21,502 transcripts, belonging to 18,935 genes (Table 3). This is comparable to the only other available Biblidinae genome assembly, which identified 21,588 transcripts (Pham et al. 2025). Likewise, both genomes contained a similar number of genes (*C. acontius*: 18,935 and *B. hypochlora*: 19,395). However, the mean length of genes was markedly different, with *B. hypochlora* having a mean length of around half of *Catonephele acontius* (*C. acontius:* 8255 bp and *B. hypochlora:* 4423.8 bp). Mean gene length in the Monarch butterfly, *Danaus plexippus,* is more similar to our *Catonephele acontius* gene lengths (9624 bp) (Gu et al. 2019) suggesting that there may have been differences in gene model annotation for *B. hypochlora* or contractions in gene length the *B. hypochlora* lineage. A BUSCO protein completeness score of 95.9% indicates high annotation completeness, with low duplication (1.6%), fragmentation (1.2%), and missing (2.8%) scores (Table 4).

**Table 3.** Gene prediction summary statistics, as computed with AGAT v.1.4.3.

Annotation statistics	*C. acontius*
Total repeat sequence masked (Mbp)	234.4
Total genes	18,935.0
Number of transcripts	21,502.0
Mean gene length (bp)	8255.0
Mean transcript size (bp)	8214.0
Gene density (per Mb)	35.9
% genome covered by genes	30.1
Total single exon genes	4791.0
Total introns in coding sequence	86,773.0
Longest gene length (bp)	241,003.0
Mean exon length (bp)	226.0
Longest exon length (bp)	22,124.0
Mean intron length in coding sequence (bp)	1517.0
Longest intron length in coding sequence (bp)	91,121.0

**Table 4.** Full BUSCO summary for final genome annotation.

Complete BUSCOs (%)	Single copy BUSCOs (%)	Duplicated BUSCOs (%)	Fragmented BUSCOs (%)	Missing BUSCOs (%)
95.9	94.3	1.6	1.2	2.8

BUSCO scores were calculated on the longest gene isoforms against the Lepidoptera gene set (*n* = 5193) with BUSCO v5.8.2 in protein mode (Manni et al. 2021).

Our combined use of long-read HiFi PacBio sequencing for HMW DNA, as well as long read IsoSeq for RNA from tissues with disparate expression profiles ensured high-quality polishing and annotation across the genome as well as high resolution for repetitive regions of the genome with limited expression. Similarly, the genome for *B. hypochlora* used Hifi and IsoSeq (from one tissue) sequencing as well as short read Illumina sequencing from a second individual. Both approaches have achieved high levels of assembly and genome annotation.

Functional annotation across the different gene prediction databases was consistently of 60% with a rise to ∼70% using Panther gene families, due to their broader classification (Supplementary Table 7).

### Chromosome number evolution

Within the tribe Epicaliini of the Biblidinae subfamily, the chromosome numbers of multiple *Catonephele* species have been established through karyotype analysis, ranging from 14 to 23. *Catonephele numilia* has 15 chromosomes (De Lesse 1970) while no counts have previously been reported for *C. acontius*. Our speculative estimate for *C. acontius* (24 contigs) (based on contig length visualization in Supplementary Fig. 2) is slightly larger than previous estimates for this genus (14–23) (Brown et al. 2007; Challis et al. 2023). However, a telomere-to-telomere analysis found very short telomeric repeat regions (<100 repeats of AACCCT) on both ends of 34 of the *C. acontius* contigs (Supplementary Table 10). All contigs without any telomeres were small, with the largest being 76Kb. We would expect 100 + repeats to give confidence in a telomere-to-telomere chromosome, based on results across the insect phylogeny (Lukhtanov and Pazhenkova 2023). As butterflies have holocentric chromosomes (Pazhenkova and Lukhtanov 2023), we do not expect a single centromeric region per chromosome. The contrast between the chromosome number estimated based on size and that estimate by telomere presence highlights that we cannot reliably determine chromosome number from this assembly. Future work on chromosome numbers in these taxa should include Hi-C or cytological analyses.

### Broad-scale genome comparison with *Melitaea cinxia* (Nymphalidae)

**Fig. 6.**
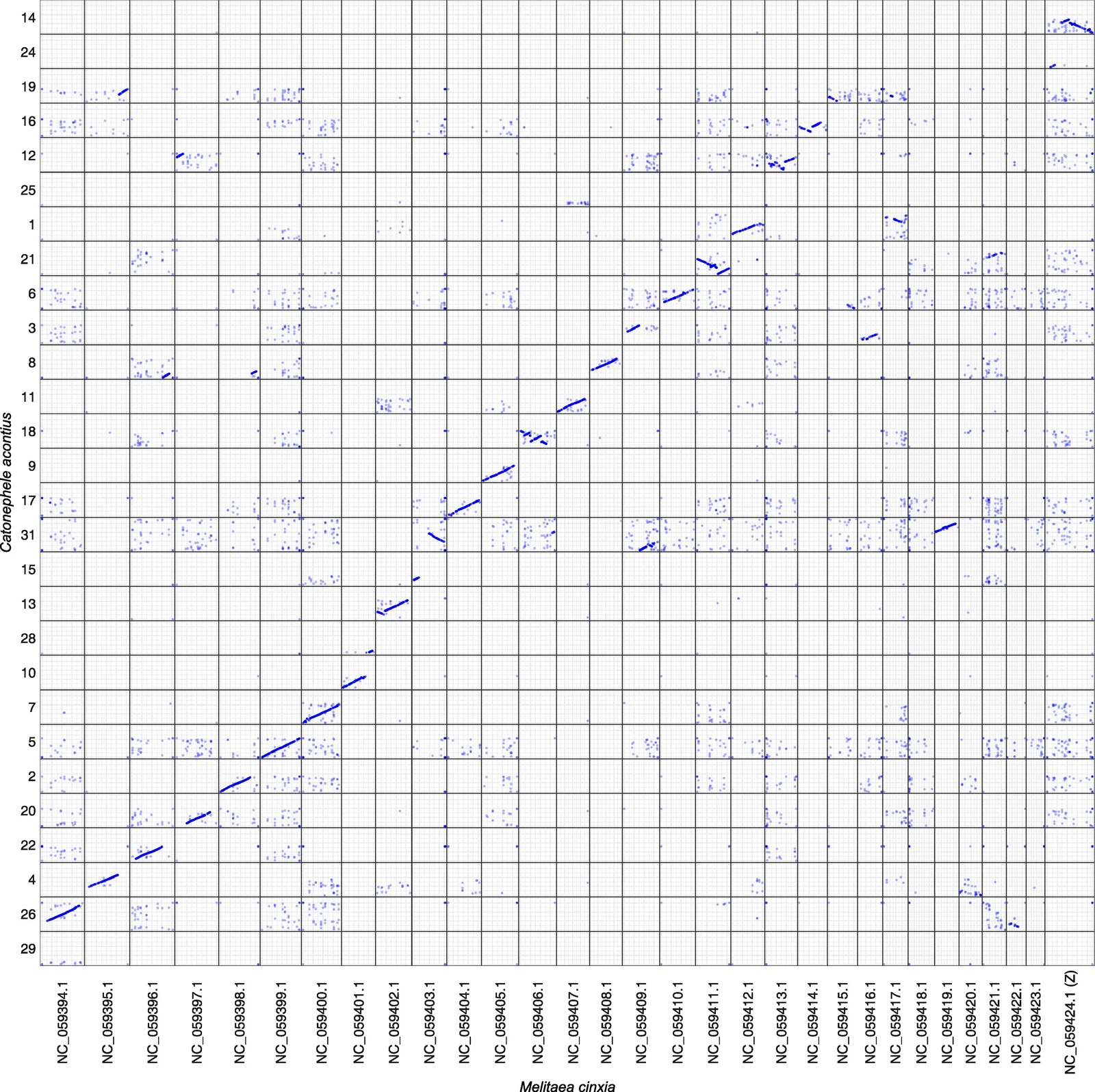
Conserved synteny between *catonephele acontius* and the outgroup nymphalidae butterfly *melitaea cinxia*. Contigs are numbered by original scaffold number to ease comparison and description. For visualization, we filtered alignments by size (<1000 bp) and contigs by size (<1 Mbp), meaning 12 contigs of *C. acontius* are not shown. The *M. cinxia* sex chromosome (Z) is NC_059424.1 (far right).

We used the *Melitaea cinxia* genome (MelCinx1.1) (Vila et al. 2021; Kumar et al. 2022) as an outgroup to study potential large-scale genome rearrangement, illustrated by a dotplot (Fig. 6). These clades diverged 56MYA (Espeland et al. 2018). We found that broad-scale genome structure was conserved across these 2 species, which was expected because, in Lepidoptera, gene order synteny is generally conserved, even if in some taxa chromosome number is more dynamic (D’Alençon et al. 2010; Ahola et al. 2014; Wright et al. 2024). We found evidence of correspondence between the sex (Z) chromosome of *M. cinxia* (NC_059424.1) and contigs 14 and 24 in *C. acontius* (Fig. 6), indicating that these contigs likely belong to the Z chromosome. These findings are supported by our coverage analysis via BlobToolKit that found these contigs have approximately half of the coverage of the other large contigs (22 and 20 × in these contigs compared to 39 × genome-wide), which would be expected as our sample was female (ZW). Further, we identified 2 small contigs (<300kbp) with approximately half of the coverage expected: contigs 36 and 45 (both 22x). Neither aligned well with an *M. cinxia* chromosome, but as MelCinx1.1 is male (ZZ), this may be because contigs 36 and 45 are W-linked. We may also have missed many other small W-linked contigs due to this limitation.

### Potential implications

Here, we generate and present a high-coverage long-read reference genome for *C. acontius,* providing a valuable genomic resource for future studies on Biblidinae butterflies. This species joins other Neotropical butterflies that have had their DNA sequenced to a high-quality scaffold-level assembly, with *B. hypochlora* as the only other genome available in Biblidinae, a subfamily found across Central and South America (Pham et al. 2025). There is recent and growing interest in the Biblidinae subfamily, including in *Panacea prola, B. hypochlora* (Pham et al. 2025) and *Catonephele* species (Hicks et al. 2026). The resources presented here therefore provide a foundational tool for comparative genomics and other techniques, such as transcriptomics and epigenomics, to understand micro- and macro-evolutionary patterns and processes in this subfamily. For example, the *Catonephele* species are being used as a case study for understanding the evolution of traits that benefit insects living in seasonal environments (Hicks et al. 2026). To date, analyses in this Nymphalidae subfamily have been largely limited to phenotypic studies. The newly assembled and annotated genomes of *C. acontius* and *B. hypochlora* will provide a foundation for investigating the genomic basis of trait evolution and ultimately inform the potential for future responses to changing environments in the wild.

## Supplementary Material

jkag174_Supplementary_Data

## Data Availability

The raw sequence data and corresponding metadata supporting the results of this article are available in the European Nucleotide Archive (ENA) at EMBL-EBI under accession number PRJEB86113, sample SAMEA117752281. The *Catonephele acontius* whole genome shotgun (WGS) project has the NCBI project accession JBYBQP000000000 (BioProject PRJNA1265232, BioSample SAMN48590164). The version described in this paper is (01) and has the accession number JBYBQP010000000. For submission to NCBI, 262 single-exon gene annotations were removed from the genome as they overlapped the exon region of another, larger gene which was flagged automatically by the system. The original genome assembly and associated gtf annotation file (without the aforementioned annotation removal) have also been uploaded to the GSA Figshare (DOI: https://doi.org/10.25387/g3.31379740) in association with this manuscript—named as follows: “acontius_reference-genome.fasta” and “SP_Acontius_annotation_v2_longest.gtf.” The pipeline for quality control, assembly, polishing, decontamination and annotation is uploaded on Figshare, as well as an “NCBI_README.txt’ file that explains exactly how the genome annotation was filtered for upload to NCBI, DOI: https://doi.org/10.6084/m9.figshare.30217813 (Hicks et al. 2025). Supplemental material available at G3 online.

## References

[jkag174-B1] Abrusán G, Grundmann N, Demester L, Makalowski W. 2009. TEclass--a tool for automated classification of unknown eukaryotic transposable elements. Bioinformatics. 25:1329–1330. 10.1093/BIOINFORMATICS/BTP084. 19349283

[jkag174-B2] Ahola V et al 2014. The Glanville fritillary genome retains an ancient karyotype and reveals selective chromosomal fusions in Lepidoptera. Nat Commun. 5:4737. 10.1038/ncomms5737. 25189940 PMC4164777

[jkag174-B3] Al Arab M et al 2017. Accurate annotation of protein-coding genes in mitochondrial genomes. Mol Phylogenet Evol. 106:209–216. 10.1016/j.ympev.2016.09.024. 27693569

[jkag174-B4] Alberto Muyshondt AM . 1975. Notes on the life cycle and natural history of butterflies of El Salvador. I B.: Hamadryas februa (Nymphalidae-Hamadryadinae). J N Y Entomol Soc. 83:157–169.

[jkag174-B5] Allio R et al 2020. MitoFinder: efficient automated large-scale extraction of mitogenomic data in target enrichment phylogenomics. Mol Ecol Resour. 20:892–905. 10.1111/1755-0998.13160. 32243090 PMC7497042

[jkag174-B6] Bache SM, Wickham H. 2022. A Forward-Pipe Operator for R [R package version 2.0.3] https://github.com/tidyverse/magrittr.

[jkag174-B7] Bao W, Kojima KK, Kohany O. 2015. Repbase update, a database of repetitive elements in eukaryotic genomes. Mob DNA. 6:11 10.1186/S13100-015-0041-9. 26045719 PMC4455052

[jkag174-B8] Beccaloni GW, Viloria Á, Hall SK, Robinson GS. 2008. Catalogue of the Hostplants of the Neotropical Butterflies/Catálogo de las Plantas Huésped de las Mariposas Neotropicales Evolution View project Seasonal cycles of the diversity and population structure of diurnal butterflies in neotropical landscapes. (Issue January).

[jkag174-B9] Bloch NI et al 2021. Different mating contexts lead to extensive rewiring of female brain coexpression networks in the guppy. Genes Brain Behav. 20:. 10.1111/GBB.12697. 32875689

[jkag174-B10] Boutet E, Lieberherr D, Tognolli M, Schneider M, Bairoch A. 2007. UniProtKB/Swiss-prot. Methods Mol Biol. 406:89–112. 10.1007/978-1-59745-535-0_4. 18287689

[jkag174-B11] Brown KS et al 2007. Chromosomal evolution in the South American nymphalidae. Hereditas. 144:137–148. 10.1111/J.2007.0018-0661.02015.X. 17850598

[jkag174-B12] Brůna T, Lomsadze A, Borodovsky M. 2020. GeneMark-EP+: eukaryotic gene prediction with self-training in the space of genes and proteins. NAR Genom Bioinform. 2:lqaa026. 10.1093/NARGAB/LQAA026. 32440658 PMC7222226

[jkag174-B13] Bryant DM et al 2017. A tissue-mapped axolotl De Novo transcriptome enables identification of limb regeneration factors. Cell Rep. 18:762–776. 10.1016/J.CELREP.2016.12.063. 28099853 PMC5419050

[jkag174-B14] Buchfink B, Xie C, Huson DH. 2015. Fast and sensitive protein alignment using DIAMOND. Nat Methods. 12:59–60. 10.1038/nmeth.3176. 25402007

[jkag174-B15] Bushnell B . 2014. BBMap: a fast, accurate, splice-aware aligner. https://sourceforge.net/projects/bbmap/.

[jkag174-B16] Camacho C et al 2009. BLAST+: architecture and applications. BMC Bioinformatics. 10:421. 10.1186/1471-2105-10-421. 20003500 PMC2803857

[jkag174-B17] Challis R, Kumar S, Sotero-Caio C, Brown M, Blaxter M. 2023. Genomes on a tree (GoaT): a versatile, scalable search engine for genomic and sequencing project metadata across the eukaryotic tree of life. Wellcome Open Res. 8:24. 10.12688/WELLCOMEOPENRES.18658.1. 36864925 PMC9971660

[jkag174-B18] Challis R, Richards E, Rajan J, Cochrane G, Blaxter M. 2020. BlobToolKit—interactive quality assessment of genome assemblies. G3 (Bethesda). 10:1361. 10.1534/G3.119.400908. 32071071 PMC7144090

[jkag174-B19] Cheng H, Concepcion GT, Feng X, Zhang H, Li H. 2021. Haplotype-resolved de novo assembly using phased assembly graphs with hifiasm. Nat Methods. 18:170–175. 10.1038/s41592-020-01056-5. 33526886 PMC7961889

[jkag174-B20] Christmas MJ et al 2023. Evolutionary constraint and innovation across hundreds of placental mammals. Science. 380:eabn3943. 10.1126/science.abn3943. 37104599 PMC10250106

[jkag174-B21] Conant GC, Wolfe KH. 2008. Genomevx: simple web-based creation of editable circular chromosome maps. Bioinformatics. 24:861–862. 10.1093/BIOINFORMATICS/BTM598. 18227121

[jkag174-B22] Cousins T, Scally A, Durbin R. 2025. A structured coalescent model reveals deep ancestral structure shared by all modern humans. Nat Genet. 57:856–864. 10.1038/s41588-025-02117-1. 40102687 PMC11985351

[jkag174-B23] Dainat J . 2024. AGAT: Another Gff Analysis Toolkit to handle annotations in any GTF/GFF format. [accessed 2024 Oct 10]. 10.5281/zenodo.3552717.

[jkag174-B24] d’Alençon E et al 2010. Extensive synteny conservation of holocentric chromosomes in lepidoptera despite high rates of local genome rearrangements. Proc Natl Acad Sci U S A. 107:7680–7685. 10.1073/PNAS.0910413107. 20388903 PMC2867904

[jkag174-B25] Danecek P et al 2021. Twelve years of SAMtools and BCFtools. GigaScience. 10:1–4. 10.1093/GIGASCIENCE/GIAB008. PMC7931819 33590861

[jkag174-B26] De Coster W, Rademakers R. 2023. NanoPack2: population-scale evaluation of long-read sequencing data. *Bioinformatics*. 39:btad311. 10.1093/bioinformatics/btad311. PMC10196664 37171891

[jkag174-B27] De Lesse H . 1970. Les nombres de chromosomes chez les lépidoptères rhopalocères en amérique centrale et colombie. Ann Soc Entomol. 6:347–358. 10.1080/21686351.1970.12277929.

[jkag174-B28] Donath A et al 2019. Improved annotation of protein-coding genes boundaries in metazoan mitochondrial genomes. Nucleic Acids Res. 47:10543–10552. 10.1093/nar/gkz833. 31584075 PMC6847864

[jkag174-B29] Espeland M et al 2018. A comprehensive and dated phylogenomic analysis of butterflies. Curr Biol. 28:770–778.e5. 10.1016/j.cub.2018.01.061. 29456146

[jkag174-B30] Flynn JM et al 2020. RepeatModeler2 for automated genomic discovery of transposable element families. Proc Natl Acad Sci U S A. 117:9451–9457. 10.1073/PNAS.1921046117. 32300014 PMC7196820

[jkag174-B31] Gabriel L et al 2024. BRAKER3: fully automated genome annotation using RNA-Seq and protein evidence with GeneMark-ETP, AUGUSTUS, and TSEBRA. Genome Res. 34:769–777. 10.1101/GR.278090.123. 38866550 PMC11216308

[jkag174-B32] Gabriel L, Hoff KJ, Brůna T, Borodovsky M, Stanke M. 2021. TSEBRA: transcript selector for BRAKER. BMC Bioinformatics. 22:566. 10.1186/S12859-021-04482-0. 34823473 PMC8620231

[jkag174-B33] García-Berro A et al 2023. Migratory behaviour is positively associated with genetic diversity in butterflies. Mol Ecol. 32:560–574. 10.1111/MEC.16770. 36336800 PMC10100375

[jkag174-B34] Gonzalez DIS . 2024. Estudio de los estadios inmaduros de dos especies lepidopteras: Catonephele acontius y Catonephele numila. I. E. Francisco Torres Leon Puente Amarillo, Colombia Presented at VIII ELEN Encuentro Internacional de Lepidoptera Neotropicales.

[jkag174-B35] Gu L et al 2019. Dichotomy of dosage compensation along the neo Z chromosome of the monarch butterfly. Curr Biol. 29:4071–4077.e3. 10.1016/j.cub.2019.09.056. 31735674 PMC6901105

[jkag174-B36] Hicks M et al 2026. Evolution of reproductive plasticity in a seasonal tropical environment. Ecol Lett. 29:e70401. 10.1111/ELE.70401. 42179311

[jkag174-B37] Hicks M, Pham TN, Seudre O. 2025. Genome assembly and annotation pipeline for a long read, high-coverage reference genome of the Nymphalid butterfly *Catonephele acontius*. *FigShare*. 10.6084/m9.figshare.30217813. PMC13535366 42391505

[jkag174-B38] King T, Butcher S, Zalewski L. 2017. Apocrita—High Performance Computing Cluster for Queen Mary University of London. 10.5281/ZENODO.438045.

[jkag174-B39] Kriventseva EV et al 2019. OrthoDB v10: sampling the diversity of animal, plant, fungal, protist, bacterial and viral genomes for evolutionary and functional annotations of orthologs. Nucleic Acids Res. 47:D807–D811. 10.1093/NAR/GKY1053. 30395283 PMC6323947

[jkag174-B40] Kumar S et al 2022. TimeTree 5: an expanded resource for Species divergence times. Mol Biol Evol. 39:msac174. 10.1093/MOLBEV/MSAC174. 35932227 PMC9400175

[jkag174-B41] Lavoie CA, Platt RN, Novick PA, Counterman BA, Ray DA. 2013. Transposable element evolution in heliconius suggests genome diversity within lepidoptera. Mob DNA. 4:21. 10.1186/1759-8753-4-21. 24088337 PMC4016481

[jkag174-B42] Li H et al 2009. The sequence alignment/map format and SAMtools. Bioinformatics. 25:2078–2079. 10.1093/BIOINFORMATICS/BTP352. 19505943 PMC2723002

[jkag174-B43] Li H . 2018. Minimap2: pairwise alignment for nucleotide sequences. Bioinformatics. 34:3094–3100. 10.1093/BIOINFORMATICS/BTY191. 29750242 PMC6137996

[jkag174-B44] Lin Y et al 2023. Quartet: a telomere-to-telomere toolkit for gap-free genome assembly and centromeric repeat identification. Hortic Res. 10:uhad127. 10.1093/HR/UHAD127. 37560017 PMC10407605

[jkag174-B45] Lukhtanov VA, Pazhenkova EA. 2023. Diversity and evolution of telomeric motifs and telomere DNA organization in insects. Biol J Linn Soc Lond. 140:536–555. 10.1093/BIOLINNEAN/BLAD068.

[jkag174-B46] Makałowski W, Gotea V, Pande A, Makałowska I. 2019. Transposable elements: classification, identification, and their use as a tool for comparative genomics. Methods Mol Biol. 1910:177–207. 10.1007/978-1-4939-9074-0_6. 31278665

[jkag174-B47] Manni M, Berkeley MR, Seppey M, Simão FA, Zdobnov EM. 2021. BUSCO update: novel and streamlined workflows along with broader and deeper phylogenetic coverage for scoring of eukaryotic, prokaryotic, and viral genomes. Mol Biol Evol. 38:4647–4654. 10.1093/MOLBEV/MSAB199. 34320186 PMC8476166

[jkag174-B48] Marçais Guillaume, Kingsford Carl. 2011. A fast, lock-free approach for efficient parallel counting of occurrences of *k* -mers. Bioinformatics. 27:764–770. 10.1093/bioinformatics/btr011. 21217122 PMC3051319

[jkag174-B49] Martín-Zamora FM et al 2023. Annelid functional genomics reveal the origins of bilaterian life cycles. Nature. 615:105–110. 10.1038/s41586-022-05636-7. 36697830 PMC9977687

[jkag174-B50] Moggioli G et al 2023. Distinct genomic routes underlie transitions to specialised symbiotic lifestyles in deep-sea annelid worms. Nat Commun. 14:2814. 10.1038/s41467-023-38521-6. 37198188 PMC10192322

[jkag174-B51] Mora P et al 2024. Sex-biased gene content is associated with sex chromosome turnover in danaini butterflies. Mol Ecol. 33:e17256. 10.1111/mec.17256. 38180347 PMC11628659

[jkag174-B52] Myers E . 2021. FASTK. https://github.com/thegenemyers/FASTK.

[jkag174-B53] Pazhenkova EA, Lukhtanov VA. 2023. Whole-genome analysis reveals the dynamic evolution of holocentric chromosomes in satyrine butterflies. Genes (Basel). 14:437. 10.3390/GENES14020437. 36833364 PMC9956908

[jkag174-B54] Pertea G, Pertea M. 2020. GFF utilities: gffRead and GffCompare. F1000Res. 9:304. 10.12688/f1000research.23297.1. PMC7222033 32489650

[jkag174-B55] Pham NT et al 2025. A high-quality draft genome assembly of the neotropical butterfly, Batesia hypochlora (nymphalidae: biblidinae). BMC Genomics. 27:31. 10.1186/S12864-025-12394-Z. 41350619 PMC12797539

[jkag174-B56] Potter SC et al 2018. HMMER web server: 2018 update. Nucleic Acids Res. 46:W200–W204. 10.1093/NAR/GKY448. 29905871 PMC6030962

[jkag174-B57] Quinlan AR . 2014. BEDTools: the Swiss-army tool for genome feature analysis. Curr Protoc Bioinformatics. 47:11.12.1–11.12.34. 10.1002/0471250953.BI1112S47. PMC4213956 25199790

[jkag174-B58] Ranallo-Benavidez TR, Jaron KS, Schatz MC. 2020. GenomeScope 2.0 and smudgeplot for reference-free profiling of polyploid genomes. Nat Commun. 11:1432. 10.1038/s41467-020-14998-3. 32188846 PMC7080791

[jkag174-B59] Ray DA et al 2019. Simultaneous TE analysis of 19 heliconiine butterflies yields novel insights into rapid TE-based genome diversification and multiple SINE births and deaths. Genome Biol Evol. 11:2162–2177. 10.1093/GBE/EVZ125. 31214686 PMC6685494

[jkag174-B60] R Core Team . 2021. R: a language and environment for statistical computing. R Foundation for Statistical Computing.

[jkag174-B61] Rosenberg NA, Nordborg M. 2002. Genealogical trees, coalescent theory and the analysis of genetic polymorphisms. Nat Rev Genet. 3:380–390. 10.1038/nrg795. 11988763

[jkag174-B62] Ruggieri AA et al 2022. A butterfly pan-genome reveals that a large amount of structural variation underlies the evolution of chromatin accessibility. Genome Res. 32:1862–1875. 10.1101/GR.276839.122. 36109150 PMC9712634

[jkag174-B63] Seppey M, Manni M, Zdobnov EM. 2019. BUSCO: assessing genome assembly and annotation completeness. Methods Mol Biol. 1962:227–245. 10.1007/978-1-4939-9173-0_14. 31020564

[jkag174-B64] Shirey V et al 2022. LepTraits 1.0 A globally comprehensive dataset of butterfly traits. Sci Data. 9:382. 10.1038/S41597-022-01473-5. 35794183 PMC9259668

[jkag174-B65] Smit A, Hubley R, Green P. 2015. RepeatMasker Open-4.0. https://www.repeatmasker.org/.

[jkag174-B66] Smolander O-P et al 2022. Improved chromosome-level genome assembly of the glanville fritillary butterfly (melitaea cinxia) integrating Pacific biosciences long reads and a high-density linkage map. GigaScience. 11:1–12. 10.1093/GIGASCIENCE/GIAB097. PMC8756199 35022701

[jkag174-B67] Stanke M, Schöffmann O, Morgenstern B, Waack S. 2006. Gene prediction in eukaryotes with a generalized hidden Markov model that uses hints from external sources. BMC Bioinformatics. 7:62. 10.1186/1471-2105-7-62. 16469098 PMC1409804

[jkag174-B68] Steward RA, de Jong MA, Oostra V, Wheat CW. 2022. Alternative splicing in seasonal plasticity and the potential for adaptation to environmental change. Nat Commun. 13:755. 10.1038/s41467-022-28306-8. 35136048 PMC8825856

[jkag174-B69] Teufel F et al 2022. Signalp 6.0 predicts all five types of signal peptides using protein language models. Nat Biotechnol. 40:1023–1025. 10.1038/s41587-021-01156-3. 34980915 PMC9287161

[jkag174-B70] Thomas PD et al 2003. PANTHER: a library of protein families and subfamilies indexed by function. Genome Res. 13:2129–2141. 10.1101/GR.772403. 12952881 PMC403709

[jkag174-B71] Uliano-Silva M et al 2023. Mitohifi: a python pipeline for mitochondrial genome assembly from PacBio high fidelity reads. BMC Bioinformatics. 24:288. 10.1186/s12859-023-05385-y. 37464285 PMC10354987

[jkag174-B72] Vila R, Hayward A, Lohse K, Wright C. 2021. The genome sequence of the glanville fritillary, *Melitaea cinxia* (Linnaeus, 1758). Wellcome Open Res. 6:266. 10.12688/WELLCOMEOPENRES.17283.1. 36873711 PMC9975429

[jkag174-B73] Vurture GW et al 2017. GenomeScope: fast reference-free genome profiling from short reads. Bioinformatics. 33:2202–2204. 10.1093/BIOINFORMATICS/BTX153. 28369201 PMC5870704

[jkag174-B74] Walker BJ et al 2014. Pilon: an integrated tool for comprehensive microbial variant detection and genome assembly improvement. PLoS One. 9:e112963. 10.1371/JOURNAL.PONE.0112963. 25409509 PMC4237348

[jkag174-B75] Warren AD, Davis KJ, Grishin NV, Pelham JP, Stangeland EM. 2012. Interactive Listing of American Butterflies. [30-XII-12]. [accessed 2025 May 10]. http://www.butterfliesofamerica.com/.

[jkag174-B76] Wenger AM et al 2019. Accurate circular consensus long-read sequencing improves variant detection and assembly of a human genome. Nat Biotechnol. 37:1155–1162. 10.1038/s41587-019-0217-9. 31406327 PMC6776680

[jkag174-B77] Wickham H . 2016. ggplot2: Elegant Graphics for Data Analysis. Springer-Verlag. ISBN 978-3-319-24277-4. https://ggplot2.tidyverse.org.

[jkag174-B78] Wickham H . 2025. stringr: Simple, Consistent Wrappers for Common String Operations. R package version 1.6.0. [accessed 2025 Dec 11 Dec]. https://stringr.tidyverse.org.

[jkag174-B79] Wickham H, François R, Henry L, Müller K, Vaughan D. 2014. dplyr: A Grammar of Data Manipulation. R package version 1.1.4 CRAN: Contributed Packages. 10.32614/CRAN.PACKAGE.DPLYR.

[jkag174-B80] Wickham H, Vaughan D, Girlich M. 2024. Tidy Messy Data [R package tidyr version 1.3.1]. CRAN: Contributed Packages. 10.32614/CRAN.PACKAGE.TIDYR. https://github.com/tidyverse/tidyr.

[jkag174-B81] Wong WY, Simakov O. 2019. RepeatCraft: a meta-pipeline for repetitive element de-fragmentation and annotation. Bioinformatics. 35:1051–1052. 10.1093/BIOINFORMATICS/BTY745. 30165587 PMC6419915

[jkag174-B82] Wright CJ et al 2025. Project psyche: reference genomes for all lepidoptera in Europe. Trends Ecol Evol. 40:1234–1250. 10.1016/j.tree.2025.10.007. 41309387

[jkag174-B83] Wright CJ, Stevens L, Mackintosh A, Lawniczak M, Blaxter M. 2024. Comparative genomics reveals the dynamics of chromosome evolution in lepidoptera. Nat Ecol Evol. 8:777–790. 10.1038/s41559-024-02329-4. 38383850 PMC11009112

[jkag174-B84] Xie Y . 2014. knitr: A comprehensive tool for reproducible research in R, Implementing Reproducible Computational Research. Chapman and Hall/CRC. https://www.taylorfrancis.com/chapters/edit/10.1201/9781315373461-1/knitr-comprehensive-tool-reproducible-research-yihui-xie.

[jkag174-B85] Xu Z, Wang H. 2007. LTR_FINDER: an efficient tool for the prediction of full-length LTR retrotransposons. Nucleic Acids Res. 35:W265–W268. 10.1093/NAR/GKM286. 17485477 PMC1933203

[jkag174-B86] Zhang Z, Schwartz S, Wagner L, Miller W. 2000. A greedy algorithm for aligning DNA sequences. J Comput Biol. 7:203–214. 10.1089/10665270050081478. 10890397

