# Supplementary material for: Long-read, high-coverage reference genome of the nymphalid butterfly Catonephele acontius (Nymphalidae: Biblidinae)
Source: G3 (Bethesda). 2026 Jul 2;16(9):jkag174. doi: 10.1093/g3journal/jkag174 (PMC13535366; doi:10.1093/g3journal/jkag174)
Supplement: Supplementary file 1 [file jkag174_supplementary_data.zip › Supplemental_Material_Legends_G3-2026-406642.docx]

**Supplementary Table 1**: Sample names and respective original tissue.

**Supplementary Table 2:** Full genome assembly statistics*.*

**Supplementary Table 3:** Full base content statistics.

**Supplementary Table 4:** Mapping rate of IsoSeq transcripts against the final genome assembly.

**Supplementary Table 5**: Repetitive element summary statistics, as generated by RepeatMasker v.4.1.7 (Smit et al., 2015). Acronyms can be found in the ‘Abbreviations’ section.

**Supplementary Table 6:** Gene counts and BUSCO score across filtering steps.

**Supplementary Table 7:** Functional annotation statistics across databases.

**Supplementary Table 8**: Kmer-based genome size estimation (Mbp) using both FastK and Jellyfish, across a range of Kmer sizes (all with max coverage 1,000,000,000)

**Supplementary Table 9**: BLAST of mitochondrial contig against all other contigs.

**Supplementary Table 10**: Telomere analysis full results. ‘Chrid’ corresponds to the chromosome (contig) ID, chrlen corresponds to the contig length (bp), ‘status’ indicates whether telomeres were on both ends of the contig, or just the left/right, or neither. ‘leftnum’ indicates how many telomeric repeats were found on the left end of the contig and ‘leftdirection’ indicates which strand the telomeric repeats were found on (+/-). The same is true for the columns ‘rightnum’ and ‘rightdirection’ for the right end.

**Supplementary Table 11**: BlobToolKit contig output.

**Supplementary Figure 1:** Ventral and dorsal images of specimen FLP-149.

**Supplementary Figure 2:** Visualisation of contig lengths revealed 24 large contigs.
