# Supplementary material for: Long-read, high-coverage reference genome of the nymphalid butterfly Catonephele acontius (Nymphalidae: Biblidinae)
Source: G3 (Bethesda). 2026 Jul 2;16(9):jkag174. doi: 10.1093/g3journal/jkag174 (PMC13535366; doi:10.1093/g3journal/jkag174)
Supplement: Supplementary file 1 [file jkag174_supplementary_data.zip › Supplementary_Figure_2_G3-2026-406642.docx]

**Supplementary Figure 2** Visualisation of contig lengths revealed 24 large contigs
