# Supplementary material for: Long-read, high-coverage reference genome of the nymphalid butterfly Catonephele acontius (Nymphalidae: Biblidinae)
Source: G3 (Bethesda). 2026 Jul 2;16(9):jkag174. doi: 10.1093/g3journal/jkag174 (PMC13535366; doi:10.1093/g3journal/jkag174)
Supplement: Supplementary file 1 [file jkag174_supplementary_data.zip › Supplementary_Table_1_G3-2026-406642.docx]

**Supplementary Table 1**: Sample names and respective original tissue

| **Sample Name** | **Species** | **Tissue** |
| --- | --- | --- |
| FLP-0149 | *C. acontius* | thorax |
| 201594_06 B1 FLP-149E | *C. acontius* | butterfly reproductive tissue |
| 201594_07 B2 FLP-149L | *C. acontius* | butterfly legs |
| 201594_08 B3 FLP-149A | *C. acontius* | butterfly antennae |
| 201594_09 B4 FLP-149G | *C. acontius* | butterfly digestive tract |
