# Supplementary material for: Long-read, high-coverage reference genome of the nymphalid butterfly Catonephele acontius (Nymphalidae: Biblidinae)
Source: G3 (Bethesda). 2026 Jul 2;16(9):jkag174. doi: 10.1093/g3journal/jkag174 (PMC13535366; doi:10.1093/g3journal/jkag174)
Supplement: Supplementary file 1 [file jkag174_supplementary_data.zip › Supplementary_Table_2_G3-2026-406642.docx]

**Supplementary Table 2:** Full genome assembly statistics

| **Assembly version** | **Total number of contigs/scaffolds** | **Total size of contigs/scaffolds (Mb)** | **Main genome scaffold N/L50 (Mb)** | **Main genome scaffold N/L90 (Mb)** | **Max scaffold length (Mb)** | **Number of scaffolds > 1Mb:** | **% main genome in scaffolds > 1Mb:** |
| --- | --- | --- | --- | --- | --- | --- | --- |
| Primary (v0.1) | 90 | 520.701 | 11/19.928 | 23/13.67 | 35.071 | 31 | 99.46 |
| Polished (v0.2) | 90 | 520.207 | 11/19.927 | 23/13.625 | 35.046 | 31 | 99.37 |
| Decontaminated (v0.3) | 40 | 519.245 | 11/19.927 | 23/13.625 | 35.046 | 31 | 99.55 |
