# Supplementary material for: Long-read, high-coverage reference genome of the nymphalid butterfly Catonephele acontius (Nymphalidae: Biblidinae)
Source: G3 (Bethesda). 2026 Jul 2;16(9):jkag174. doi: 10.1093/g3journal/jkag174 (PMC13535366; doi:10.1093/g3journal/jkag174)
Supplement: Supplementary file 1 [file jkag174_supplementary_data.zip › Supplementary_Table_3_G3-2026-406642.docx]

**Supplementary Table 3:** Full base content statistics

| **Assembly version** | **A (%)** | **C (%)** | **G (%)** | **T (%)** | **GC (%)** | **GC_stdev (%)** |
| --- | --- | --- | --- | --- | --- | --- |
| *C. acontius v0.1* | 33.33 | 16.66 | 16.66 | 33.35 | 33.32 | 8.06 |
| *C. acontius v0.2* | 33.34 | 16.65 | 16.66 | 33.35 | 33.31 | 8.05 |
| *C. acontius v0.3* | 33.32 | 16.67 | 16.67 | 33.34 | 33.34 | 3.77 |
