# Supplementary material for: Long-read, high-coverage reference genome of the nymphalid butterfly Catonephele acontius (Nymphalidae: Biblidinae)
Source: G3 (Bethesda). 2026 Jul 2;16(9):jkag174. doi: 10.1093/g3journal/jkag174 (PMC13535366; doi:10.1093/g3journal/jkag174)
Supplement: Supplementary file 1 [file jkag174_supplementary_data.zip › Supplementary_Table_4_G3-2026-406642.docx]

**Supplementary Table 4:** Mapping rate of IsoSeq transcripts against the final genome assembly.

| **Library type** | **Mapping rate (%)** |
| --- | --- |
| Ovary RNA (IsoSeq) | 99.93 |
| Legs RNA (IsoSeq) | 99.86 |
| Antennae RNA (IsoSeq) | 99.93 |
| Digestive tissue RNA (IsoSeq) | 99.93 |
