# Supplementary material for: Long-read, high-coverage reference genome of the nymphalid butterfly Catonephele acontius (Nymphalidae: Biblidinae)
Source: G3 (Bethesda). 2026 Jul 2;16(9):jkag174. doi: 10.1093/g3journal/jkag174 (PMC13535366; doi:10.1093/g3journal/jkag174)
Supplement: Supplementary file 1 [file jkag174_supplementary_data.zip › Supplementary_Table_5_G3-2026-406642.docx]

**Supplementary Table 5**: Repetitive element summary statistics, as generated by RepeatMasker v.4.1.7 (Smit et al., 2015). Acronyms can be found in the ‘Abbreviations’ section.

| **Class** | ***C. acontius*** | |
| --- | --- | --- |
|  | **Total length (bp)** | **% of genome** |
| Unclassified | 12,128,736 | 2.29 |
| non-LTR | 10,701,538 | 2.02 |
| DNA transposons | 79,232,758 | 14.95 |
| LTRs | 10,957,556 | 2.07 |
| LINEs | 80,452,202 | 15.18 |
| Retroelements | 23,569,550 | 4.45 |
| SINEs | 7,508,837 | 1.42 |
| Unmasked | 294,693,669 | 55.60 |
| Simple repeats | 10,764,578 | 2.03 |

Smit, A., Hubley, R., & Green P. (2015). *RepeatMasker Open-4.0*.
