# Supplementary material for: Long-read, high-coverage reference genome of the nymphalid butterfly Catonephele acontius (Nymphalidae: Biblidinae)
Source: G3 (Bethesda). 2026 Jul 2;16(9):jkag174. doi: 10.1093/g3journal/jkag174 (PMC13535366; doi:10.1093/g3journal/jkag174)
Supplement: Supplementary file 1 [file jkag174_supplementary_data.zip › Supplementary_Table_6_G3-2026-406642.docx]

**Supplementary Table 6 –** Gene counts and BUSCO score across filtering steps. BUSCO scores were calculated on the longest gene isoforms against the Lepidoptera gene set (n=5,193) with BUSCO v.5.1.2 in protein mode (Manni et al., 2021).

| **Polishing step** | **Complete**  **BUSCOs (%)** | **Single copy**  **BUSCOs (%)** | **Duplicated**  **BUSCOs (%)** | **Fragmented**  **BUSCOs (%)** | **Missing**  **BUSCOs (%)** | **Gene counts** |
| --- | --- | --- | --- | --- | --- | --- |
| Initial | 97.2 | 84.0 | 13.2 | 1.2 | 1.6 | 22,635 |
| No gene with stop codon | 97.2 | 84.0 | 13.2 | 1.2 | 1.6 | 22,635 |
| No gene overlapping TE | 97.2 | 84.0 | 13.2 | 1.2 | 1.6 | 22,019 |
| No gene TE-like | 96.0 | 83.1 | 12.9 | 1.2 | 2.7 | 18,935 |

Manni, M., Berkeley, M. R., Seppey, M., Simão, F. A., & Zdobnov, E. M. (2021). BUSCO Update: Novel and Streamlined Workflows along with Broader and Deeper Phylogenetic Coverage for Scoring of Eukaryotic, Prokaryotic, and Viral Genomes. *Molecular Biology and Evolution*, *38*(10), 4647–4654. https://doi.org/10.1093/MOLBEV/MSAB199
