# Supplementary material for: Long-read, high-coverage reference genome of the nymphalid butterfly Catonephele acontius (Nymphalidae: Biblidinae)
Source: G3 (Bethesda). 2026 Jul 2;16(9):jkag174. doi: 10.1093/g3journal/jkag174 (PMC13535366; doi:10.1093/g3journal/jkag174)
Supplement: Supplementary file 1 [file jkag174_supplementary_data.zip › Supplementary_Table_7_G3-2026-406642.docx]

**Supplementary Table 7 –** Functional annotation statistics across databases. BLASTP and BLASTX v2.16.0 were performed against the UniProtKB/SwissProt database (Boutet et al., 2007), Pfam domain detection with HMMER v3.3 (Potter et al., 2018) and signal peptide prediction with SignalP v5.0 (Teufel et al., 2022). Panther families were assigned with PANTHER v19.0 (Thomas et al., 2003).

| **Category** | ***C. acontius* (%)** |
| --- | --- |
| BLASTX | 60.85 |
| BLASTP | 59.41 |
| Pfam | 60.60 |
| SignalP | 11.64 |
| KEGG | 51.45 |
| Gene Ontology (BLASTX) | 59.98 |
| Gene Ontology (BLASTP) | 58.64 |
| Gene Ontology (Pfam) | 36.35 |
| Panther family ID | 70.18 |

Boutet, E., Lieberherr, D., Tognolli, M., Schneider, M., & Bairoch, A. (2007). UniProtKB/Swiss-Prot. *Methods in Molecular Biology (Clifton, N.J.)*, *406*, 89–112. https://doi.org/10.1007/978-1-59745-535-0_4

Potter, S. C., Luciani, A., Eddy, S. R., Park, Y., Lopez, R., & Finn, R. D. (2018). HMMER web server: 2018 update. *Nucleic Acids Research*, *46*(W1), W200–W204. https://doi.org/10.1093/NAR/GKY448

Teufel, F., Almagro Armenteros, J. J., Johansen, A. R., Gíslason, M. H., Pihl, S. I., Tsirigos, K. D., Winther, O., Brunak, S., von Heijne, G., & Nielsen, H. (2022). SignalP 6.0 predicts all five types of signal peptides using protein language models. *Nature Biotechnology 2022 40:7*, *40*(7), 1023–1025. https://doi.org/10.1038/s41587-021-01156-3

Thomas, P. D., Campbell, M. J., Kejariwal, A., Mi, H., Karlak, B., Daverman, R., Diemer, K., Muruganujan, A., & Narechania, A. (2003). PANTHER: A Library of Protein Families and Subfamilies Indexed by Function. *Genome Research*, *13*(9), 2129–2141. https://doi.org/10.1101/GR.772403
