# Supplementary material for: Long-read, high-coverage reference genome of the nymphalid butterfly Catonephele acontius (Nymphalidae: Biblidinae)
Source: G3 (Bethesda). 2026 Jul 2;16(9):jkag174. doi: 10.1093/g3journal/jkag174 (PMC13535366; doi:10.1093/g3journal/jkag174)
Supplement: Supplementary file 1 [file jkag174_supplementary_data.zip › Supplementary_Table_8_G3-2026-406642.docx]

**Supplementary Table 8 –** Kmer-based genome size estimation (Mbp) using Jellyfish and FastK across a range of Kmer sizes (all with max coverage 1,000,000,000).

| **Software** | **k = 21** | **k = 31** | **k = 41** |
| --- | --- | --- | --- |
| FastK | 401.654 | 416.092 | 418.743 |
| Jellyfish | 434.353 | 440.032 | 439.026 |
